# 复发性胸腺瘤的治疗

**DOI:** 10.3779/j.issn.1009-3419.2020.03.11

**Published:** 2020-03-20

**Authors:** 显平 刘, 晓 李, 剑锋 李

**Affiliations:** 100044 北京，北京大学人民医院胸外科 Department of Thoracic Surgery, Peking University People's Hospital, Beijing 100044, China

**Keywords:** 复发性胸腺瘤, 手术治疗, 化疗, 放疗, 靶向治疗, Recurrent thymoma, Surgical treatment, Chemotherapy, Radiotherapy, Targeted therapy

## Abstract

胸腺瘤是一种具有恶性潜能的疾病，完整切除后的复发率为10%-30%。复发性胸腺瘤的治疗策略及标准未达成一致，治疗方式包括再次手术治疗、化疗、放疗、靶向治疗及免疫治疗等，至今存在着争议。在这篇综述中，通过回顾既往的文献，总结了复发性胸腺瘤的不同治疗方法的适应证、疗效以及预后，以期给复发性胸腺瘤治疗标准的制定提供一些参考。

## Abstract (en)

Thymoma is a potential malignant disease with a recurrence rate of 10% to 30% after complete resection. There is no agreement on the treatment strategy and standard for recurrent thymoma. The treatment methods include reoperation, chemotherapy, radiotherapy, targeted therapy and immunotherapy. In this article, we review the previous literature and summarize the indications, efficacy and prognosis of different treatments for recurrent thymoma, so as to provide some reference for treatment criteria for recurrent thymoma.

胸腺瘤是前纵隔最常见的肿瘤，起源于胸腺上皮细胞，是一种低度恶性肿瘤，据报道每年发病率为1.3%-3.2%^[1]^。完整的手术切除是胸腺瘤最有效的治疗方式，完整切除后的胸腺瘤复发率为10%-30%^[2-4]^，复发时间从术后数年到数十年不等，平均复发时间约为5年^[5]^。由于胸腺瘤复发或者进展十分缓慢，需要进行长期的随访，建议使用5年生存率和10年生存率来评估胸腺瘤患者的预后。

根据国际胸腺恶性肿瘤兴趣小组（International Thymic Malignancy Interest Group）的共识，复发性胸腺瘤指的是根治性手术切除或根治性放疗后的完全缓解患者再次出现的新病灶。胸腺瘤的复发可分为3类^[5]^：①局部复发：胸腺床部位的复发（如前纵隔、正常胸腺、胸腺瘤邻近组织中出现的新病灶）；②区域复发：即胸腔内的复发肿瘤，且不与胸腺或原胸腺肿瘤紧邻（这里主要指胸膜区域）；③远处复发：胸腔外部位及肺实质内的复发。复发性胸腺瘤血源性转移罕见（< 5%），绝大多数为胸膜区域复发，其次为局部复发^[6, 7]^。

公认的与胸腺瘤复发相关的危险因素主要包括：肿瘤Masaoka分期、世界卫生组织（World Health Organization, WHO）组织学分型、首次手术切除的彻底性（是否R0切除）^[8, 9]^。肉眼或镜下可见的胸膜或心包侵犯患者复发的风险增加，Wright等^[10]^按WHO组织学分型将胸腺瘤分为A/AB、B1/B2、B3和C，其局部侵袭性和复发率逐步上升，C型胸腺瘤的复发率远高于其他类型（41% *vs* 9.7%）^[8]^。但是新分型中C型胸腺瘤已经完全从胸腺瘤中分出，成为独立的胸腺癌。

关于复发性胸腺瘤的治疗目前尚没有统一的标准，相关文献报道数量较少，治疗的选择主要依据单中心的治疗经验。本文检索了1990年1月-2019年6月期间发表的相关文献，总结了复发性胸腺瘤的治疗选择以及相关预后，以期给予复发性胸腺瘤治疗标准的制定提供一些参考。

## 1 手术切除

### 1.1 适应证

很多研究^[1-4, 11-16]^都推荐再次手术来治疗复发性胸腺瘤，但是再次手术存在一定的技术困难和风险，因为第一次手术为了达到R0切除通常会切除所有胸腺和周边脂肪组织，导致相近区域的血管和神经暴露及黏连浸润，同时胸腔内的器官频繁受累，更易导致胸腔内播散复发^[3]^。美国国家综合癌症网络（National Comprehensive Cancer Network, NCCN）指南^[17]^指出，对于局部复发、单侧出现区域复发的胸腺瘤患者，手术可作为一种选择。

因此正确理解复发性胸腺瘤再次手术的适应证十分重要。很多文献都建议手术适应证为：局限于胸腔内（局部复发、区域复发及肺实质内的转移）的复发性胸腺瘤，经影像学评估技术上可切除（不存在广泛的胸内器官侵犯，未侵袭至大血管或心脏），能耐受手术^[1, 2, 15, 18-20]^。Bott等^[14]^通过对复发性胸腺瘤和胸腺癌的研究指出，除了满足上述条件，若病变是在单侧可考虑完整手术切除，若胸腔双侧均有复发或广泛的单侧胸腔内复发，可行减瘤手术。有部分文献^[9, 21]^报道胸膜外肺切除术可用于胸腺瘤的广泛胸膜复发，适应证为年轻、肺功能良好无术前并发症的患者，能够达到肉眼上的完整切除。Hamaji等^[8]^提出手术禁忌证包括：单侧胸膜复发伴广泛病变；双侧胸膜复发；术后早期复发；双侧肺实质复发；颈部淋巴结转移；胸腔外复发及全身情况差。

### 1.2 手术原则及手术方式

众多的研究^[2, 8]^建议手术原则是能完整切除者应尽量完整切除，若不能完整切除应最大限度切除复发的瘤体。Filippo等^[22]^指出若复发性胸腺瘤可切除，为减少其再次复发的几率，可行扩大切除术。Dai等^[23]^指出由于减瘤手术预后情况较差，一般在其他治疗手段不可行的情况下可考虑实施。

复发性胸腺瘤的手术方式分为两大类，即开放性手术和微创手术，后者主要是指电视辅助胸腔镜手术（video-assisted thoracoscopic surgery, VATS）。开放性手术（开胸手术）切口入路有3种：经胸骨正中切口、前外侧切口及腋下小切口、后外侧切口^[2]^。复发的位置及病灶数量是手术方式的决定因素，前/后外侧切口开胸术适用于胸膜、胸膜-肺、肺实质复发的患者，当复发局限于纵隔时行再次胸骨正中切开术更加适宜。

文献报道的手术方式主要以开放性手术为主，其中以前/后外侧切口开胸术为主，Bott等^[14]^的研究统计，复发性胸腺瘤的手术方式中前/后外侧切口开胸术占72.7%、胸骨正中切开术占9.1%，胸骨部分切开术占9.1%。Marulli等^[15]^回顾性分析了4个医学中心1980年-2010年的数据，行手术治疗的复发性胸腺瘤患者中，前/后外侧切口开胸术52例（71.2%），胸骨正中切开术13例（17.8%），VATS 3例。

近年来，各种新的技术及微创手术被越来越多地应用于复发性胸腺瘤患者的手术治疗中。Marulli等^[24]^认为复发性胸腺瘤行VATS的最佳指征是单侧胸膜/肺复发和单侧纵隔非侵袭性复发，因为这种情况下行肺楔形切除术或胸膜切除术安全且有效，创伤小，术后恢复快。Tseng等^[25]^在胸腺瘤胸腺复发再次手术时应用了氩离子凝固术，证明了其安全性、有效性和可行性，但是需要后续更进一步的研究论证。Abtin等^[26]^报道使用经皮冷冻切除术来治疗纵隔及胸腹复发的胸腺瘤患者，随访近1年的时间中，90%的病灶无局部复发。但是该研究随访时间过短，对于预后良好的胸腺瘤而言，1年的随访结果显然是不够的。

### 1.3 预后

多项研究的结果表明，再次手术的患者仍有良好的疗效（表 1）。总体来看，首次手术后复发的中位时间为48个月-93个月，说明胸腺瘤进展缓慢，需要长期随访。除Haniduda等^[27]^的研究报道复发性胸腺瘤R0切除率 < 30%之外，其他研究者的R0切除率为45%-90%，能够达到比较满意的比例。总体的5年和10年生存率分别为37%-85%、16%-74%，R0切除复发性胸腺瘤的患者5年和10年生存率分别为64%-91%、47%-91%，R0切除组患者5年和10年生存率明显高于总体患者。因此，多项研究^[2-4, 9, 11, 18, 19, 28, 29]^均建议复发性胸腺瘤行手术治疗，若条件允许应行完整切除（R0），完整切除的患者有更高的生存率。

**1:** 复发性胸腺瘤的再手术与预后 Reoperation and prognosis of recurrent thymoma

Author	Year	Median RFI (mo)	Total (*N*)	Reoperation (*n*)	R0 resection rate (%)	5-yr OS (%)			10-yr OS (%)	
						Total	R0 resection		Total	R0 resection
Urgesi^[28]^	1992	-	30	11	45	85	-		74	-
Regnard^[2]^	1997	88	28	28	67	51	64		43	54
Ruffini^[3]^	1997	86	30	16	63	48	72		24	72
Haniduda^[27]^	2001	-	24	15	27	37	-		16	-
Vita^[11]^	2007	88	20	20	-	43	-		37	-
Okumura^[36]^	2007	-	67	22	82	-	-		70	-
Lucchi^[12]^	2009	60	20	20	65	43	-		26	-
Margaritora^[18]^	2011	93	43	30	73	64	91		51	67
Hamaji^[8]^	2012	61	30	20	90	54	-		-	-
Bae^[31]^	2012	52	41	15	87	60	91		33	91
Sandri^[29]^	2014	-	81	61	73	69	82		52	65
Mizuno^[30]^	2015	-	237	115	-	76	-		50	-
Marulli^[15]^	2016	50	103	73	69	63	80		37	60
Fiorelli^[16]^	2017	-	53	38	84	52	72		32	47
RFI: recurrence-free interval; OS: overall survival.										

关于复发胸腺瘤手术与非手术组预后的比较目前缺乏前瞻性数据，多中心回顾性研究具有更大的参考价值。Mizuno等^[30]^回顾性收集了全日本1991年-2010年期间通过手术治疗的胸腺上皮肿瘤（包含胸腺瘤和胸腺癌）患者的医疗数据，初次切除后复发的患者420例，其中复发的胸腺瘤患者243例，其5年和10年生存率分别为76.2%和50.0%，总体上手术组的生存期显着优于非手术组。Sandri等^[29]^回顾性分析了3个医学中心2001年1月-2013年6月的数据，共计81例复发性胸腺瘤患者，61例给予再手术治疗，其中45例R0切除；其余20例行非手术治疗（放疗±化疗或姑息治疗），结果显示总体5年生存率为68.7%，10年生存率为52%，手术组与非手术组的5年和10年生存率分别为70.2% *vs* 54.1%和64.3% *vs* 46.9%，R0切除组与非R0切除组相比5年生存率（70.2% *vs* 64.3%）和10年生存率（54.1% *vs* 46.9%）均更高。说明手术治疗对于复发性胸腺瘤有明显的效果，且R0切除预后更好。Fiorelli及其同事^[16]^回顾性研究了4个医学中心1984年-2014年共计515例胸腺瘤患者，53例患者复发，其中38例行手术治疗，15例行非手术治疗（3例化疗，10例放化疗，2例支持治疗）。在多因素回归分析中，完整切除是唯一显著的预后因子，完全切除组的生存率高于不完全切除组或无手术治疗组（*P*=0.02）。Hamaji及其同事^[4]^纳入1991年-2013年发表的11项回顾性研究中278例复发性胸腺瘤患者的治疗结果，R0切除率为45%-91%（平均值：67.2%±20.4%），手术治疗与非手术治疗（放疗/化疗）相比有着更好的5年生存率（70.9%±16.2% *vs* 49.6%±27.4%）和10年生存率（29.6%±21.9% *vs* 18.4%±26%），手术治疗可明显改善长期生存，延长患者的寿命。

但是由于所有的研究都是回顾性的，进行再次手术的患者存在明显的选择偏倚：患者病情局限、身体状态良好，本来就存在更好的生存预期^[13, 22]^，据报道^[23]^，复发胸腺瘤再次手术时，手术死亡率为0.0%-13.3%，手术并发症发生率为0.0%-32.1%。这可能是大部分研究中手术治疗的患者预后明显优于非手术治疗患者的原因之一，因此需要更好匹配的回顾性研究或者前瞻性随机对照试验来进行验证。

### 1.4 术后辅助治疗

复发胸腺瘤再手术后的辅助治疗也十分重要，因为既往的研究数据表明再次手术的根治性切除率有限，再次术后仍有27%-50%的患者会出现再次复发。理论上再次复发术后的辅助治疗应该能改善疾病的局部和系统控制率^[16]^。辅助治疗包括化疗、放疗，其中最常使用的是放疗。

然而，复发后再次手术患者中行术后辅助治疗的比例并不高，Hamaji及其同事的*meta*分析^[4]^指出，辅助化疗和放疗的比例很低，分别占再手术患者的20.4%±32.7%和20.9%±32.8%。Bae等^[31]^报道再次手术后行辅助治疗可一定程度上防止再次复发。Fiorelli等^[16]^的研究表明复发性胸腺瘤根治性切除（R0）后的辅助治疗能够显著改善其预后。

对于胸膜复发的胸腺瘤患者，有部分研究^[12, 29, 32]^推荐在手术切除后进一步行胸腔热灌注化疗，可有效防止胸腺瘤的再次复发。胸腔热灌注化疗的方案有多柔比星+顺铂、丝裂霉素+顺铂，化疗药物通过胸引管灌入胸腔内，温度维持在41 ℃-43 ℃，持续60 min，Ambrogi等^[32]^的研究报道手术后行胸腔内热灌注5年生存率为92%，Maury等^[33]^的结果表示复发性胸腺瘤患者的中位无病生存期为42个月，能够提供长期的局部控制，在特定情况下可延长患者的生存期。

术后辅助治疗作为复发性胸腺瘤多模式综合治疗中的一环，能够在一定程度上防止再次复发，而对于改善预后生存还仍有争议。

## 2 放疗

很多研究认为，经评估后不可切除的局部或区域复发、不能耐受手术的胸腺瘤患者，特别是广泛的胸膜复发，放疗是一种有效的治疗方法。一项回顾性研究^[28]^中获得了较高的反应率，仅接受根治性放疗的复发性胸腺瘤患者7年生存率达65%。Ruffini等^[3]^的研究中有11例复发性胸腺瘤患者仅接受了放疗，放疗方案为剂量总量38 Gy-44 Gy、每次1.8 Gy-2 Gy，持续4周-5周，但单纯放疗组生存率低于手术治疗组。有文献^[6]^报道低剂量半胸放疗被证实安全且可有助于控制胸膜转移/复发的胸腺瘤患者。Margaritora等^[18]^的研究中不适合手术切除的复发性胸腺瘤患者行放疗和/或化疗，其5年生存率仅为35%，而手术组患者为77%。Sandri等^[29]^多中心回顾性研究结果表明单纯接受化学/放射治疗的患者的5年和10生存率分别为72.4%和55.7%。单纯放疗在复发性胸腺瘤中应用较少，且相关的生存数据及研究也较少，但放疗作为一种有效的治疗方式在复发性胸腺瘤的治疗中仍有很重要的地位。

## 3 化疗

许多文献^[1, 31]^报道化疗可作为不可切除的复发性胸腺瘤的首要选择。根据已有的文献^[6, 12, 31]^，化疗的方案几乎都是以铂类为基础，顺铂和蒽环类药物最常见，方案包括环磷酰胺+多柔比星+顺铂（CAP）、顺铂+多柔比星+环磷酰胺+长春新碱（ADOC）、顺铂+依托泊苷+表阿霉素（EPA）、卡铂+紫杉醇（TC）、卡铂+多柔比星（TA）。除此之外，有文献^[20, 34]^报道培美曲塞、吉西他滨、5-氟尿嘧啶、亮氨酸等也可用于复发性胸腺瘤的全身化疗。

已接受蒽环类药物治疗的患者中，顺铂/卡铂-依托泊苷是胸腺瘤首次复发最常用的方案，卡铂-紫杉醇则是比较常见的三线化疗方案^[20]^。为了降低全身化疗的毒性并实现更好的局部控制，Terada及其同事^[35]^通过肋间动脉和膈下动脉输注化疗药物（50 mg顺铂+20 mg多柔比星），成功治疗了2例胸膜复发胸腺瘤患者。但该化疗给药途径报道的病例数较少，其效果及副反应需进一步评估。

Sandri等^[29]^的研究表明，放疗/化疗的复发性胸腺瘤患者5年和10年生存率分别为64.3%、46.9%。部分研究^[18, 36]^结果显示，单纯给予放疗/化疗复发性胸腺瘤患者的5年生存率可 > 35%。因此不可行手术治疗的患者，也应给予积极的治疗来延缓病情发展和延长患者生存时间。

## 4 靶向治疗及免疫治疗

近年来，关于复发性胸腺瘤靶向治疗的研究越来越多，也积累了一些经验。胸腺瘤细胞中表皮生长因子受体（epidermal growth factor receptor, EGFR）高度表达，但突变少见，因此酪氨酸激酶抑制剂（tyrosine kinase inhibitor, TKI）可能无效^[37, 38]^。有病例报告称*EGFR*突变的复发患者对吉非替尼无反应^[39]^。然而，一些EGFR强阳性的胸腺瘤患者给予西妥昔单抗治疗后得到部分缓解^[40, 41]^。另一项研究^[42]^显示贝伐珠单抗与厄洛替尼联合治疗多次复发的胸腺瘤患者，未观察到肿瘤反应，但60%的患者病情稳定。一篇来自*Lancet Oncology*的II期临床研究显示西妥木单抗治疗难治性或复发性胸腺瘤患者，14%的患者获得部分缓解，76%患者病情稳定^[43]^。此外，有报道^[44]^显示使用贝利司他（组蛋白去乙酰酶抑制剂）治疗难治性晚期胸腺瘤患者，8%的患者获得部分缓解，2年总生存率为77%。

生长抑素受体在胸腺上皮肿瘤等多种恶性肿瘤中均有表达，奥曲肽是一种强效生长抑素类似物，在体外有抑制胸腺上皮细胞的作用，因此奥曲肽被认为是复发性胸腺瘤的一种治疗选择^[45, 46]^。奥曲肽联合醋酸泼尼松治疗复发性胸腺瘤患者时，28.5%-30.3%的患者表现出疾病应答，35.7%-36.8%的患者病情稳定^[40]^。最近有II期临床试验^[47]^结果显示，长效奥曲肽联合醋酸泼尼松用于局部复发不可切除的恶性胸腺瘤的新辅助治疗，88%的患者有治疗反应，53%的患者治疗后成功接受了手术治疗。

研究显示，胸腺瘤中存在*c-KIT*基因突变，给予铂类化疗失败的难治性晚期胸腺瘤患者舒尼替尼治疗，一项临床II期研究中^[48]^，16例患者中有1例部分缓解、12例病情稳定，疾病控制率达81%。除此之外，一项来自法国RYTHMIC网络的28例难治性胸腺癌及胸腺瘤患者的回顾性研究^[49]^中，胸腺瘤患者的疾病控制率为86%，缓解率为29%，中位无病生存期为5.4个月，证明舒尼替尼在此类患者中有效。另有报道^[50, 51]^称索拉非尼也是治疗胸腺瘤的有效药物，但相关的病例报告较少。

经铂类化疗后进展的晚期/复发性胸腺瘤或胸腺癌患者，一项II期临床试验^[52]^使用依维莫司（mTOR抑制剂）治疗，32例胸腺瘤患者中，3例完全缓解、27例病情稳定，疾病控制率达93.8%，中位无进展生存期为16.6个月，证明依维莫司可诱导持久的疾病控制。

目前有研究报道了PD-1/PD-L1抑制剂在晚期胸腺上皮肿瘤中的应用。一项II期临床试验^[53]^使用PD-1受体的完全人源化IgG4抗体派姆单抗（Pembrolizumab）治疗胸腺癌，41例患者中有6例（15%）发生严重的自身免疫性疾病，缓解率达到了23%，其中完全缓解1例，部分缓解8例，病情稳定21例（53%），中位无进展生存期和总生存期分别为4.2个月和24.9个月。在韩国也进行了类似的研究^[54]^，共计33例难治性或复发性胸腺上皮瘤患者，其中胸腺瘤患者7例，5例（71%）病情稳定、2例（29%）部分缓解，但有5例（71%）患者出现了三级以上的免疫相关不良事件。一项来自日本的II期试验^[55]^，使用纳武单抗（Nivolumab）（PD-1受体的IgG4抗体）治疗胸腺癌患者，第一阶段共纳入15例不可切除或复发性胸腺癌患者，11例患者病情稳定，中位无进展生存期为3.8个月。总体而言，晚期胸腺瘤患者进行免疫治疗不良反应率高，且缓解率偏低。

近年来关于复发性胸腺瘤靶向治疗及免疫治疗的研究越来越多，给我们提供了不同的治疗思路。

## 5 结语

总体来看，若胸腺瘤复发范围局限、身体状况能耐受手术，仍建议再次手术治疗，可明显延长生存期，再次术后是否进一步行辅助治疗仍存在争议。若复发病变范围较广泛，可采用化疗、姑息性放疗或放化疗，仍可延长生存期，但其远期生存预后不如再次手术组，且毒副作用较多。部分化疗/放疗效果不明显的患者，还可选择靶向治疗或免疫治疗，但针对复发性胸腺瘤的相关研究较少，疗效值得商榷。

复发性胸腺瘤是一种不多见的疾病，其进展缓慢、异质性明显，多学科的综合治疗方案效果评价存在一定的困难。关于复发性胸腺瘤治疗的争议仍然存在，大多数研究者仍然推荐以手术治疗为主的综合治疗。但仍需大样本的多中心合作前瞻性临床试验为制定复发性胸腺瘤的治疗策略提供可靠的依据。

## References

[1] Yokoi K, Kondo K, Fujimoto K (2017). JLCS medical practice guidelines for thymic tumors: summary of recommendations. Jpn J Clin Oncol.

[2] Regnard JF, Zinzindohoue F, Magdeleinat P (1997). Results of re-resection for recurrent thymomas. Ann Thorac Surg.

[3] Ruffini E, Mancuso M, Oliaro A (1997). Recurrence of thymoma: analysis of clinicopathologic features, treatment, and outcome. J Thorac Cardiovasc Surg.

[4] Hamaji M, Ali SO, Burt BM (2014). A *meta*-analysis of surgical versus nonsurgical management of recurrent thymoma. Ann Thorac Surg.

[5] Huang J, Detterbeck FC, Wang Z (2010). Standard outcome measures for thymic malignancies. J Thorac Oncol.

[6] Lucchi M, Basolo F, Mussi A (2008). Surgical treatment of pleural recurrence from thymoma. Eur J Cardiothorac Surg.

[7] Ciccone AM, Rendina EA (2005). Treatment of recurrent thymic tumors. Semin Thorac Cardiovasc Surg.

[8] Hamaji M, Allen MS, Cassivi SD (2012). The role of surgical management in recurrent thymic tumors. Ann Thorac Surg.

[9] Ruffini E, Filosso PL, Oliaro A (2009). The role of surgery in recurrent thymic tumors. Thorac Surg Clin.

[10] Wright CD, Wain JC, Wong DR (2005). Predictors of recurrence in thymic tumors: importance of invasion, World Health Organization histology, and size. J Thorac Cardiovasc Surg.

[11] Vita ML, Tessitore A, Cusumano G (2007). Recurrence of thymoma: re-operation and outcome. Ann Ital Chir.

[12] Lucchi M, Davini F, Ricciardi R (2009). Management of pleural recurrence after curative resection of thymoma. J Thorac Cardiovasc Surg.

[13] Lucchi M, Mussi A (2010). Surgical treatment of recurrent thymomas. J Thorac Oncol.

[14] Bott MJ, Wang H, Travis W (2011). Management and outcomes of relapse after treatment for thymoma and thymic carcinoma. Ann Thorac Surg.

[15] Marulli G, Margaritora S, Lucchi M (2016). Surgical treatment of recurrent thymoma: is it worthwhile?. Eur J Cardiothorac Surg.

[16] Fiorelli A, D'Andrilli A, Vanni C (2017). Iterative surgical treatment for repeated recurrences after complete resection of thymic tumors. Ann Thorac Surg.

[17] Ettinger DS, Riely GJ, Akerley W (2013). Thymomas and thymic carcinomas: Clinical Practice Guidelines in Oncology. J Natl Compr Canc Netw.

[18] Margaritora S, Cesario A, Cusumano G (2011). Single-centre 40-year results of redo operation for recurrent thymomas. Eur J Cardiothorac Surg.

[19] Park B, Park JS, Kim HK (2011). Surgical management of locoregionally recurrent thymoma. Thorac Cancer.

[20] Banna GL, Sheel A, Sheel V (2017). Treatment and prognostic factors of patients with thymic epithelial tumors at first recurrence or progression. Future Oncol.

[21] Shiiya H, Hida Y, Kaga K (2018). Extrapleural pneumonectomy of recurrent thymoma with pleural dissemination. Respirol Case Rep.

[22] Filippo L, Alfredo C, Stefano M (2012). Caveats in recurrent thymoma management. J Thorac Oncol.

[23] Dai J, Song N, Yang Y (2015). Is it valuable and safe to perform reoperation for recurrent thymoma?. Interact Cardiovasc Thorac Surg.

[24] Marulli G, Comacchio GM, Rea F (2015). Video assisted thoracic surgery (VATS) for recurrent thymoma. Ann Cardiothorac Surg.

[25] Tseng YC, Tseng YH, Tang EK (2018). A new application for argon beam coagulation: the thymoma patient with pleural recurrence. J Thorac Dis.

[26] Abtin F, Suh RD, Nasehi L (2015). Percutaneous cryoablation for the treatment of recurrent thymoma: preliminary safety and efficacy. J Vasc Interv Radiol.

[27] Haniuda M, Kondo R, Numanami H (2001). Recurrence of thymoma: clinicopathological features, re-operation, and outcome. J Surg Oncol.

[28] Urgesi A, Monetti U, Rossi G (1992). Aggressive treatment of intrathoracic recurrences of thymoma. Radiother Oncol.

[29] Sandri A, Cusumano G, Lococo F (2014). Long-term results after treatment for recurrent thymoma: a multicenter analysis. J Thorac Oncol.

[30] Mizuno T, Okumura M, Asamura H (2015). Surgical management of recurrent thymic epithelial tumors: a retrospective analysis based on the Japanese nationwide database. J Thorac Oncol.

[31] Bae MK, Byun CS, Lee CY (2012). Clinical outcomes and prognosis of recurrent thymoma management. J Thorac Oncol.

[32] Ambrogi MC, Korasidis S, Lucchi M (2016). Pleural recurrence of thymoma: surgical resection followed by hyperthermic intrathoracic perfusion chemotherapydagger. Eur J Cardiothorac Surg.

[33] Maury JM, Girard N, Tabutin M (2017). Intra-thoracic chemo-hyperthermia for pleural recurrence of thymoma. Lung Cancer.

[34] Gbolahan OB, Porter RF, Salter JT (2018). A phase II study of pemetrexed in patients with recurrent thymoma and thymic carcinoma. J Thorac Oncol.

[35] Terada Y, Kambayashi T, Okahashi S (2005). Trans-arterial infusion chemotherapy for recurrence of pleural dissemination after thymectomy. Ann Thorac Surg.

[36] Okumura M, Shiono H, Inoue M (2007). Outcome of surgical treatment for recurrent thymic epithelial tumors with reference to world health organization histologic classification system. J Surg Oncol.

[37] Suzuki E, Sasaki H, Kawano O (2006). Expression and mutation statuses of epidermal growth factor receptor in thymic epithelial tumors. Jpn J Clin Oncol.

[38] Karube Y, Kobayashi S, Maeda S (2016). Tumor-related gene expression levels in thymic carcinoma and type B3 thymoma. J Cardiothorac Surg.

[39] Nakagiri T, Funaki S, Kadota Y (2014). Does gefitinib have effects on EGFR mutation-positive thymoma? -Case report of thymoma recurrence. Ann Thorac Cardiovasc Surg.

[40] Palmieri G, Marino M, Salvatore M (2007). Cetuximab is an active treatment of metastatic and chemorefractory thymoma. Front Biosci.

[41] Farina G, Garassino MC, Gambacorta M (2007). Response of thymoma to cetuximab. Lancet Oncol.

[42] Azad A, Herbertson RA, Pook D (2009). Motesanib diphosphate (AMG 706), an oral angiogenesis inhibitor, demonstrates clinical efficacy in advanced thymoma. Acta Oncol.

[43] Rajan A, Carter CA, Berman A (2014). Cixutumumab for patients with recurrent or refractory advanced thymic epithelial tumours: a multicentre, open-label, phase 2 trial. Lancet Oncol.

[44] Giaccone G, Rajan A, Berman A (2011). Phase II study of belinostat in patients with recurrent or refractory advanced thymic epithelial tumors. J Clin Oncol.

[45] Krenning EP, Kwekkeboom DJ, Bakker WH (1993). Somatostatin receptor scintigraphy with [^111^In-DTPA-D-Phe1]- and [^123^I-Tyr3]- octreotide: the Rotterdam experience with more than 1,000 patients. Eur J Nucl Med.

[46] Ferone D, van Hagen PM, van Koetsveld PM (1999). *In vitro* characterization of somatostatin receptors in the human thymus and effects of somatostatin and octreotide on cultured thymic epithelial cells. Endocrinology.

[47] Kirzinger L, Boy S, Marienhagen J (2016). Octreotide LAR and prednisone as neoadjuvant treatment in patients with primary or locally recurrent unresectable thymic tumors: a phase II study. PLoS One.

[48] Thomas A, Rajan A, Berman A (2015). Sunitinib in patients with chemotherapy-refractory thymoma and thymic carcinoma: an open-label phase 2 trial. Lancet Oncol.

[49] Remon J, Girard N, Mazieres J (2016). Sunitinib in patients with advanced thymic malignancies: Cohort from the French RYTHMIC network. Lung Cancer.

[50] Bolzacchini E, Chini C, Pinotti G (2016). Response of malignant thymoma to sorafenib. J Thorac Oncol.

[51] Du J, Zhou XJ (2017). Precise diagnosis and treatment of thymic epithelial tumors based on molecular biomarkers. Crit Rev Oncog.

[52] Zucali PA, De Pas T, Palmieri G (2018). Phase II study of everolimus in patients with thymoma and thymic carcinoma previously treated with cisplatin-based chemotherapy. J Clin Oncol.

[53] Giaccone G, Kim C, Thompson J (2018). Pembrolizumab in patients with thymic carcinoma: a single-arm, single-centre, phase 2 study. Lancet Oncol.

[54] Cho J, Kim HS, Ku BM (2019). Pembrolizumab for patients with refractory or relapsed thymic epithelial tumor: an open-label phase II trial. J Clin Oncol.

[55] Katsuya Y, Horinouchi H, Seto T (2019). Single-arm, multicentre, phase II trial of nivolumab for unresectable or recurrent thymic carcinoma: PRIMER study. Eur J Cancer.

